# Modified reporting of positive urine cultures to reduce treatment of asymptomatic bacteriuria in long-term care facilities: a randomized controlled trial

**DOI:** 10.1093/jacamr/dlac109

**Published:** 2022-10-14

**Authors:** Zahra Rehan, Claire Pratt, Kim Babb, Brenda Filier, Laura Gilbert, Robert Wilson, Peter Daley

**Affiliations:** Memorial University, Room 1J421, 300 Prince Phillip Dr, A1B 3V6, St. John’s, NL, Canada; Memorial University, Room 1J421, 300 Prince Phillip Dr, A1B 3V6, St. John’s, NL, Canada; Memorial University, Room 1J421, 300 Prince Phillip Dr, A1B 3V6, St. John’s, NL, Canada; Eastern Health Region, St. John’s, NL, Canada; Eastern Health Region, St. John’s, NL, Canada; Public Health Microbiology Laboratory, St. John’s, NL, Canada; Quality of Care NL, St. John’s, NL, Canada; Memorial University, Room 1J421, 300 Prince Phillip Dr, A1B 3V6, St. John’s, NL, Canada; Eastern Health Region, St. John’s, NL, Canada

## Abstract

**Objectives:**

We conducted a prospective, randomized, unblinded superiority trial of the safety and efficacy of modified reporting of positive urine cultures to improve the appropriateness of treatment for asymptomatic bacteriuria (ASB) and urinary tract infection (UTI) in long-term care facilities (LTCFs).

**Methods:**

Consecutive positive urine cultures collected from LTCF patients were randomized between standard (identification and susceptibility) or modified (without identification and susceptibility) laboratory reports. Exclusion criteria were current antibiotic treatment, neutropenia, or transfer to acute care. The diagnosis of UTI or ASB was made prospectively.

**Results:**

One hundred and sixty-nine urine cultures were considered, 100 were randomized and included in ITT analysis, and 96 were included in PP analysis. Sixty-two out of 100 (62%) patients had ASB [41/62 (66%) treated] and 38/100 (38%) had UTI [35/38 (92%) treated]. The lab was called to report the identification and susceptibility in 31/51 (61%) modified reports. The rate of appropriate treatment was higher in the modified report arm: 31/51 (61%) versus 25/49 (51%) (+10%, *P* = 0.33). Untreated ASB was higher in the modified report arm: 13/32 (41%) versus 8/30 (27%) (+14%, *P* = 0.25). There were two deaths (one treated ASB, one untreated ASB) and 15 adverse events in the modified arm. There were no deaths (*P* = 0.16) and 11 adverse events (*P* = 0.43) in the standard arm. Three patients with untreated UTI survived.

**Conclusions:**

Modified reporting of urine culture improved the appropriateness of treatment by reducing treatment of ASB, but not significantly. Many LTCF prescribers requested standard urine culture reports. Modified reporting may not be suitable for LTCF implementation.

## Introduction

Asymptomatic bacteriuria (ASB) is common among elderly patients in long-term care facilities (LTCFs).^1^ Antimicrobial treatment of ASB does not prevent urinary tract infection (UTI), complications or death, and may increase antimicrobial resistance, adverse drug effects and *Clostridioides difficile*-associated diarrheoa.^2,3^ Antimicrobial use is common in LTCFs and interventions are needed to improve antimicrobial stewardship in LTCFs, including reducing the use of antimicrobials for treatment of ASB.^4^

The decision to treat ASB is often made in response to the receipt of a positive urine culture result. If the reason for collection of urine culture is unknown to the prescriber interpreting the positive culture, antibiotics may be given inappropriately for ASB. Traditional antimicrobial stewardship interventions in LTCFs, including education, clinical practice guidelines and prospective audit and feedback, do not directly address the provider’s decision to prescribe in response to the positive urine culture. Modification of the wording of the positive urine culture report may change the interpretation of the result and the consequent patient management/treatment decisions.

Laboratory-based antimicrobial stewardship interventions may improve antimicrobial treatment decisions.^5^ Modified reporting of urine cultures was first described in an observational study.^6^ We performed two randomized trials previously to evaluate modified reporting of positive urine cultures in acute care, demonstrating improvement in treatment appropriateness in the modified reporting arm.^7,8^ Appropriate treatment was defined as both treatment for UTI and lack of treatment for ASB, since the intervention is designed to reduce treatment of ASB without reducing treatment of UTI. The aim of this study was to apply this same modified reporting intervention in LTCFs, to determine if modified reporting improved the appropriateness of ASB/UTI treatment decisions.

## Materials and methods

### Trial design

The study was a prospective, randomized, parallel, unblinded superiority trial comparing two different methods of reporting for positive urine cultures. There were no changes to the trial design during the study.

### Participants

Between 5 November 2018 and 29 June 29 2019, urine specimens received for urine culture testing were inoculated onto blood and MacConkey agar plates, incubated overnight and interpreted according to the standard laboratory procedures at the Public Health Microbiology Laboratory, Division of Laboratory Medicine, Eastern Health, in St. John’s, NL, Canada. This laboratory performs all microbiology testing in the city. Urine specimens submitted from any of the eight LTCFs in the city were assessed prospectively for eligibility using clinical electronic medical records (EMR). The inclusion criteria were urine cultures demonstrating significant growth, collected by midstream or in-and-out catheterization from adult patients (aged ≥18 years) admitted to LTCFs. The exclusion criteria were urine cultures collected from an indwelling catheter, patients not residing in a LTCF (transferred to acute care), pregnancy, initiation of antibiotic treatment at the time of collection, or neutropenia.

### Intervention

Physicians in LTCFs were notified about the trial before initiation. Eligible urine cultures were randomized to standard reporting (SR) or modified reporting (MR), prior to reporting in the EMR system. The SR included bacterial count, identification, and antimicrobial susceptibility. The MR withheld this information and stated: ‘This POSITIVE urine culture may represent asymptomatic bacteriuria or urinary tract infection. If urinary tract infection is suspected clinically, please call the microbiology laboratory at (phone number) between 09:00 and 23:00, or the microbiology technologist on-call at (phone number) at night, for identification and susceptibility results’. If a provider contacted the laboratory and requested the SR, the SR was released immediately by telephone and updated in the EMR system. Participants were followed for 30 days after reporting to monitor adverse events and safety outcomes, using the EMR system and paper charts.

After culture reporting, the clinical diagnosis of UTI or ASB was determined at 72 h by a geriatrician investigator, using the EMR and completing an assessment using published criteria.^2^ UTI was defined as one or more of: fever (>38.0°C), suprapubic tenderness, costovertebral angle pain or tenderness, increase in urine frequency, increase in urinary urgency, and dysuria. ASB was defined as positive urine culture in the absence of any of these signs or symptoms. Participants were reassessed at 7 days and 30 days by a geriatrician to collect treatment data and study outcomes. There was no communication between study investigators and attending physicians. If participants were discharged during the 30 day follow-up period, health records were reviewed, and the primary care physician was contacted.

### Outcomes

The primary efficacy outcome was the proportion of appropriate antibiotic treatment, defined as treated UTI and untreated ASB. The secondary efficacy outcome was the proportion of requests for SR in the MR arm. A request for SR after receiving MR represents a failure of the MR report to inform the provider’s treatment decision alone, so a request for SR in the MR arm was considered a failure of the MR intervention. Safety was reported as mortality or bacteraemia or other adverse event rate over 30 days. Adverse events were defined using the systemic inflammatory response syndrome (SIRS) criteria (two or more of: temperature >38.3°C or <36°C, heart rate >90/minute, respiratory rate >20/minute, and leucocyte count >12 000 or <4000/mm^3^, altered mental status, significant oedema, and hyperglycaemia in the absence of diabetes), or any other new symptom or unscheduled healthcare visit recorded in the medical record.

### Sample size

The effect size was estimated from our previous study, which reported an increase in appropriateness of treatment from 29/55 (52.7%) in the SR to 44/55 (80.0%) in the MR.^7^ For a comparison of proportions between two equal groups, accepting a significance level of 5% and statistical power of 80%, a sample size of 2*N* = 90 participants was calculated. To account for missing data or loss to follow-up, 100 participants were recruited. There was no interim analysis.

### Randomization

A randomization sequence was generated without blocking or stratification, using Microsoft Excel, Version 1903, by an investigator not involved in enrolling participants. Allocation concealment was maintained by placing the reporting assignments into serially numbered, sealed and opaque envelopes to be opened by investigators at the time of recruitment.

### Blinding

The attending physicians and investigators were not able to be blinded to the intervention because the laboratory report revealed the intervention arm. The investigators performed the analysis.

### Statistical methods

All specimens randomized and reported were analysed using ITT analysis. Inappropriately included specimens were excluded from a PP analysis. The proportion of appropriate treatment and subgroup analyses were compared between arms using a two-sided Pearson chi-squared test (SPSS Statistics software, IBM USA).

### Ethics

The research was conducted in accordance with the Declaration of Helsinki and national and institutional standards. The protocol was approved by the Provincial Health Research Ethics Board on 16 July 2018 (file 2018.121). The requirement for patient consent was waived because physicians were the research subjects. The requirement for physician consent was waived because the intervention posed minimal risk to participants, and awareness of the study may have influenced therapy decisions. Serious adverse events were reported to the ethics committee within 24 h.

## Results

### Participant flow

**Figure 1.**
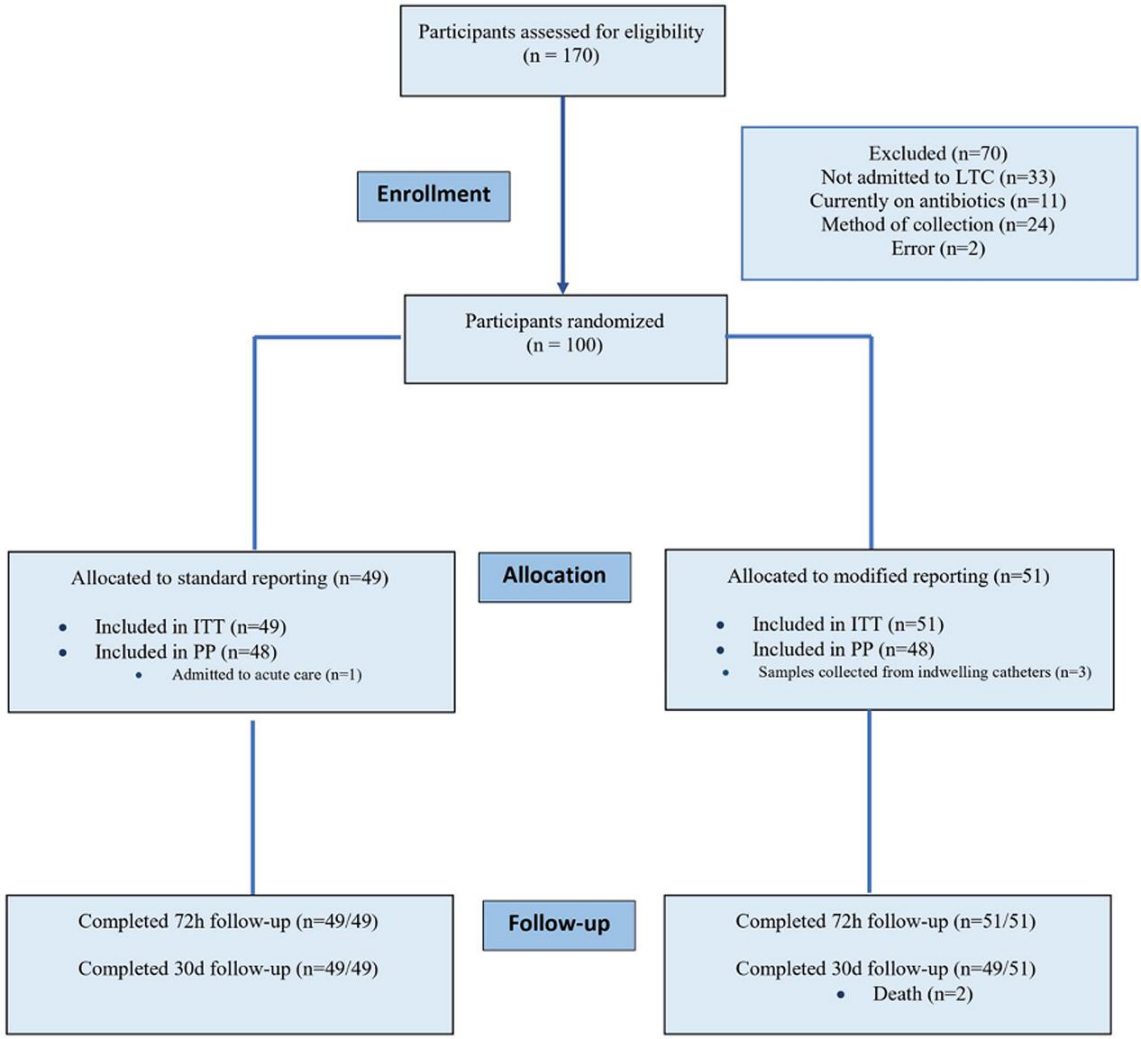
Participant flow.

One hundred and seventy consecutive positive urine culture specimens (participants) were assessed and 70/170 (41.2%) were excluded, see Figure 1. One hundred participants were randomized and included in ITT analysis: 49 randomized to the SR arm and 51 randomized to the MR arm. One patient out of 49 (2.0%) was excluded from the SR arm because the patient was admitted to acute care during the recruitment, and 3/51 (5.9%) were excluded from the MR arm because urines were collected from indwelling catheters. PP analysis was performed on remaining participants. Forty-nine of 49 (100%) participants in the SR arm, and 49/51 (96.1%) in the MR arm completed 30 day follow-up. All participants were analysed in originally assigned groups.

### Recruitment

The trial ended when the sample size was recruited.

### Baseline data

The two groups were comparable, see Table 1, with similar mean age (SR 74.0 ± 17.7 years, MR 76.1 ± 12.5 years) and proportion of females (SR 71.4%, MR 66.7%). Thirty-eight of one hundred (38.0%) were diagnosed with UTI and 62/100 (62.0%) were diagnosed with ASB. The proportion of UTI (SR 38.8%, MR 37.3%) and ASB (SR 61.2%, MR 62.7%) were similar in both arms. Seventy-six of 100 (76.0%) were treated with antibiotics; 41/62 (66.1%) of ASB were treated and 35/38 (92.1%) of UTI were treated.

**Table 1.** Patient demographics

	SR (*n* = 49)	MR (*n* = 51)
Age, years (mean ± SD)	74.0 ± 17.7	76.1 ± 12.5
Females, *n* (%)	35 (71.4)	34 (66.7)
UTI, *n* (%)	19 (38.8)	19 (37.3)
ASB, *n* (%)	30 (61.2)	32 (62.7)

### Outcomes

The rate of appropriate treatment was non-significantly higher in the MR arm compared with the SR arm (31/51 (60.8%) versus 25/49 (51.0%), absolute difference = 9.8%, relative risk (RR) = 1.19, 95% CI (0.83–1.69), see Table 2. Findings were similar in the ITT and PP populations.

**Table 2.** Appropriate treatment rate

	SR	MR	Absolute risk difference	RR (95% CI)
ITT population, *n*/*N* (%)	25/49 (51.0)	31/51 (60.8)	+9.8%	1.19 (0.84–1.69)
PP population, *n*/*N* (%)	24/48 (50.0)	30/48 (62.5)	+12.5%	1.25 (0.87–1.79)

Among 16/51 (31.4%) participants randomized to the MR arm, healthcare workers called the laboratory requesting SR. Among participants who crossed over to the SR arm, appropriateness of treatment worsened non-significantly [16/29 (55.2%) versus 15/22 (68.2%), absolute difference = −13.0%, RR = 0.81, 95% CI (0.52–1.25)], see Table 3.

**Table 3.** Appropriate treatment rate MR arm

	MR Arm		Absolute risk difference	RR (95% CI)
	Request for SR	No Request for SR		
ITT population, *n*/*N* (%)	16/29 (55.2)	15/22 (68.2)	−13.0%	0.81 (0.52–1.25)
PP population, *n*/*N* (%)	15/28 (53.6)	15/20 (75.0)	−21.4%	0.71 (0.47–1.10)

MR had a significantly stronger impact on appropriate treatment among patients with ASB compared with patients with UTI (absolute difference = 13.9% versus 5.2%, *P* = 0.048), see Table 4.

**Table 4.** Appropriate treatment rate, subgroups

Diagnosis	SR (%)	MR (%)	Absolute risk difference	RR (95% CI)
ITT population				
* *UTI (*N* = 38), *n* (%)	17/19 (89.5)	18/19 (94.7)	+5.2%	1.06 (0.87–1.28)
* *ASB (*N* = 62), *n* (%)	8/30 (26.7)	13/32 (40.6)	+13.9%	1.57 (0.76–3.26)
PP population				
UTI (*N* = 38), *n* (%)	17/19 (89.5%)	18/19 (94.7)	+5.2%	1.06 (0.87–1.28)
ASB (*N* = 62), *n* (%)	7/29 (24.1)	12/29 (41.4)	+17.3%	1.71 (0.79–3.73)

### Safety

There were complete safety data available at 72 h on all patients. There were complete safety data available at 30 days for 98/100 (98.0%) patients (two deaths during follow-up). There were no deaths in the SR arm. There were two deaths in the MR arm (one untreated ASB and one treated ASB). Both deaths were not considered related to the study intervention, see Table 5.

**Table 5.** Deaths

Study number	48	49
Study arm	MR	MR
Age	82	85
Gender	Female	Male
Reason for admission	Clinical decline	COPD exacerbation, Pneumonia, decreased level of consciousness
Reason for urine culture collection	Unknown	Unknown
Urine culture date	11 February 2019	12 February 2019
Blood culture date and result	None	None
Study diagnosis	ASB treated	ASB untreated
Study day of death	4	26
Antimicrobial therapy	Septra DS	None
Presumed cause of death	Decline	Congestive heart failure
Death related to intervention	No	No

There was one bacteraemia in the SR arm and no bacteraemia in the MR arm. The bacteraemia occurred on Day 22 of the follow-up period. Because the patient received treatment for ASB, the bacteraemia was not considered related to the study intervention.

At 72 h and at 30 days, SIRS occurred more frequently in the MR arm [SR 6/14 (42.8%), MR 8/14 (57.1%), *P* = 0.45 at 72 h; SR 27/61 (44.2%), MR 34/61 (55.7%), *P* = 0.20 at 30 days], see Tables 6 and 7. SIRS at 72 h may have been associated with the initial presentation, but SIRS at 30 days was considered related to the study intervention. Adverse events at 30 days were more frequent in the MR arm compared with the SR arm [SR 27/61 (44.2%), MR 34/61 (55.7%), *P* = 0.20]. None of the safety outcomes demonstrated a significant difference between study arms.

**Table 6.** Adverse events over 72 h

	MR	SR
Tachycardia, *n*	5	1
Abnormal temperature, *n*	2	1
Hyperglycaemia, *n*	0	0
Oedema, *n*	0	0
Elevated WBC count, *n*	1	1
Altered mental status, *n*	0	3
Tachypnoea, *n*	0	0

**Table 7.** Adverse events over 30 days

	MR	SR
Tachycardia, *n*	14	8
Abnormal temperature, *n*	8	5
Hyperglycaemia, *n*	0	1
Oedema, *n*	1	0
Elevated WBC count, *n*	3	4
Altered mental status, *n*	5	4
Tachypnoea, *n*	3	5

There were three cases of untreated UTI in the MR arm, but these did not result in adverse events or mortality over 30 days.

## Discussion

We hypothesized that MR would improve the appropriateness of treatment in LTCFs. We demonstrated that MR was associated with a non-significant increase in the appropriateness of treatment, without a significant reduction in safety. Although we failed to achieve a significant difference, we measured indicators for future quality improvement. The intention of MR is to influence the interpretation of the positive urine culture away from a decision to treat. The MR does not change the provider’s beliefs about the significance of the urine culture result. For this reason, MR may have less impact in influencing treatment decisions in LTCFs compared with acute care, where we previously reported a significant difference,^7^ if LTCF providers’ beliefs strongly favour treatment of ASB compared with acute care providers. Our study was powered based on the improvement observed using the same intervention in acute care hospitals, but the observed impact in LTCFs was less. Treatment decisions made by LTCF providers were not as strongly influenced by the MR. Despite the lack of statistically significant difference, we have produced evidence to inform sample size calculations for future interventions. Our intervention is a simple and feasible laboratory intervention, which does not require ongoing effort.

Sixty-two out of 100 (62%) positive urine cultures in our LTCF study represented ASB. Urine culture should not be collected from patients who do not meet UTI diagnostic criteria. The inappropriate collection of urine cultures promotes treatment of ASB and further intervention may reduce this behaviour.

In 31/51 (60.8%) of MR reports, healthcare workers called the lab to request the SR, causing significant crossover of these participants from the MR arm into the SR arm. This rate of requests for SR was higher compared with the rate of requests for SR in our previous study in acute care (33.3%).^6^ MR had a more positive influence on appropriate treatment of patients with ASB compared with UTI, indicating that ASB treatment decision is based on responding to a culture result, as opposed to clinical assessment.

MR did not cause increased death or bacteraemia among LTCF patients; however, a non-significantly higher rate of adverse events and three cases of untreated UTI were observed. The LTCF population has a high rate of mortality and acute illness. We cannot conclude that MR is safe in LTCFs based on our data.

### Limitations

We relied on EMRs and clinical data provided by LTCF nursing and physician staff to make the diagnosis of ASB or UTI and collect safety data. If medical records were incomplete, our diagnosis may have been biased towards ASB. Given the randomization, this bias would have a balanced impact in both groups. It was not possible to blind the investigators because investigators accessed final urine culture reports while assessing outcome. This lack of blinding did not influence treatment decisions, because investigators were not involved in treatment decisions; however, awareness of assignment may have influenced outcome assessment.

### Generalizability

Our inclusion criteria represented a large proportion of all urine cultures collected from LTCFs, suggesting that our findings may be generalizable to other LTCFs. We excluded urine collected from indwelling catheters because indwelling catheters are associated with a significant increase in risk of UTI.^9^

It may be worthwhile to repeat this study in LTCFs; however, we observed a high rate of requests for SR, suggesting that the MR intervention may not be accepted by LTCF providers. In LTCFs, interventions such as education and algorithms have not proven to be effective in improving urine culture ordering or rate of appropriate antimicrobial use.^10^ Urine culture restriction interventions may be more suitable in LTCFs.^11^ Access to urine culture could be restricted to specific clinical criteria indicating UTI (‘pre-authorization’). Furthermore, it may be helpful to explore provider attitudes towards UTI diagnosis and treatment in LTCFs.

### Interpretation

Treatment of ASB is common in LTCFs. Modified reporting reduced treatment of ASB, but not significantly. Many prescribers requested the SR, causing crossover of patients from the MR arm to the SR arm. Modified reporting may not be suitable for LTCF implementation without further study.

## Transparency declarations

There are no conflicts of interest to declare.

### Roles of authors

All authors made substantial contributions to: the conception or design of the work; or the acquisition, analysis, or interpretation of data for the work; and drafting the work or revising it critically for important intellectual content; and final approval of the version to be published; and agreement to be accountable for all aspects of the work in ensuring that questions related to the accuracy or integrity of any part of the work are appropriately investigated and resolved. Z.R.: investigation, data curation, analysis, writing: original draft, visualization; C.P.: investigation, data curation, writing: review and editing; K.B.: investigation, writing: review and editing; B.F.: investigation, writing: review and editing; L.G.: randomization, writing: review and editing; P.D.: conceptualization, methodology, analysis, data curation, writing: review and editing, supervision, project administration.

## Registration

Clinicaltrials.gov Identifier: NCT03554603.

## Protocol

Submitted with manuscript to journal.
